# Tools used to appraise the quality of studies included in systematic reviews and meta-analyses in human genetics: a systematic review

**DOI:** 10.1038/s41431-025-01861-6

**Published:** 2025-05-21

**Authors:** Carmen Lindsay, Ingeborg Blancquaert, François Rousseau

**Affiliations:** 1 Axe Santé des Populations et Pratiques Optimales en Santé, Centre de recherche CHU de Québec-Université Laval, Québec, QC G1L 3L5 Canada; 2 Research methodology consultant, Montréal, QC Canada; 3 Department of molecular biology, medical biochemistry and pathology, Faculty of Medicine, Université Laval, Québec, Canada; 4 Population Health and Optimal Health Practices Research Axis, Centre de recherche du CHU de Québec-Université Laval, 10 rue de l’Espinay, Québec, QC G1L 3L5 Canada

**Keywords:** Biomarkers, Medical research, Diseases

## Abstract

Quality assessment of primary studies is an essential component of systematic reviews (SRs). This methodological review systematically examines the choice, format and utilization of critical appraisal (CA) tools in SRs with or without meta-analyses in the field of human genetics. We searched MEDLINE, Embase, Web of Science, and PubMed up to January 2024. Two reviewers independently performed title, abstract, full-text screening and data extraction. This PROSPERO registered methodological review followed PRISMA guidelines. Meta-analysis and full-scale risk-of-bias assessment of SRs were not relevant. Among 149 randomly selected SRs, 136 mentioned CA tools (156 citations). Nineteen different generic tools constituted 71.2% of citations. NOS, QUADAS and the Cochrane risk-of-bias tool represented 36.5, 11.5, and 8.3% of tools, respectively. Ninety-three reviews stated following reporting guidelines, with 22 PRISMA checklists accessible. Detailed presentation of results was observed for 65.8% of generic and 37.8% of customized tools (*p* = 0.0013). Results for NOS were less often detailed than for other generic tools (*p* < 0.0001). Few SRs used CA results for study selection, data analysis, or discussion of findings. In conclusion, this first review of CA tools in human genetics SRs highlights a lack of transparency regarding utilization of CA tools and deficiencies in reporting of CA results.

Registration: PROSPERO (CRD42023449349)

## Introduction

The Human Genome Project has allowed identification of numerous genetic markers associated with disease. Some discoveries paved the way for the implementation of genetic testing in clinical settings. In both clinical and public health contexts, it is crucial to evaluate the advantages, risks, and constraints associated with utilizing genetic markers. A significant challenge lies in the scarcity of scientific evidence upon which to ground these assessments [1], and the quality of evidence needs to be improved [2]. Systematic reviews (SRs) stand out as a cornerstone of evidence-based medicine which is associated with improved health care outcomes. Unlike narrative reviews, SRs meticulously identify, appraise, and synthetize evidence from all pertinent studies meeting predefined eligibility criteria, offering a structured and comprehensive assessment [3]. By minimizing bias and ensuring reliable and valid data syntheses, SRs play a pivotal role in informing subsequent medical and public health decisions.

Even though methodological guidance regarding the proper conduct of SRs and meta-analyses exists, there is still room for improvement in terms of conduct and reporting of published SRs. Initiatives to support better reporting include the EQUATOR network and the QUOROM [4] and PRISMA statements [5], while tools such as AMSTAR/AMSTAR-2 [6] and ROBIS [7] have been proposed to assess the quality and risk of bias of SRs.

Quality assessment (QA) of primary studies is an essential component of SRs [3]. Relying solely on the study design is outdated; other quality aspects must also be considered [8–10]. Study quality often refers to internal validity, the extent to which study design, conduct, and analysis have minimized biases [11]. Structured reviews, such as health technology assessment (HTA) or comparative effectiveness research, tend to use a traditional epidemiological approach whereby research methodology is scrutinized to determine whether biases are likely and in what direction and to what extent such biases could affect the study findings. Multiple tools have been developed to standardize the QA process and most tools are design-specific, since different biases may affect different types of studies. In recent years tools have been developed which go beyond collecting factual or descriptive information and are structured around the plausibility of specific types of biases. Although the ultimate goals of most tools seem to converge, there are currently some tensions regarding the appropriateness of including in critical appraisal (CA) tools only items related to risk of bias (RoB) or also items related to methodological and reporting quality [12]. Separating items related to different concepts, such as risk of bias or applicability, has been advocated [9].

As a result of these diverse streams, there is a great variability across CA tools in terms of exhaustivity and specificity with respect to study type, clinical domain or even technology, but also in terms of complexity of use. The tools requiring judgements to be made entail a greater complexity, require more methodological competency and are susceptible to greater inter-rater variability [13]. Finally, CA tools can be conceived as scales, as checklists or as component or domain-based tools. A shift away from scales towards component-based assessments has been advocated since overall scores can lead to inconsistencies [12, 14, 15]. Authors of SRs are thus confronted with complex decisions when choosing the most appropriate CA tool. Indeed, numerous CA tools have been tallied for different study types, including among others randomized clinical trials, diagnostic accuracy studies, and observational studies [11, 15–19]. Ideally, tools should have been rigorously developed and validated, as well as being easy to use [16].

In the field of human genetics, efforts to support adequate synthesis and evaluation of the literature have been driven by initiatives such as the HuGENet Review project [20] and the EGAPP project [21] as well as within the HTA community. In terms of tools, Little and colleagues proposed a checklist to support “Reporting, Appraising, and Integrating Data on Genotype Prevalence and Gene Disease Associations” as early as 2002 [22], and in 2009, STREGA [23], an extension of STROBE [24], was proposed as a reporting guideline for genetic association studies. In 2010, the Genetic testing Evidence Tracking Tool (GETT) was developed to structure the collection of all relevant information for evaluation purposes [25]. However, it was not until 2015 that Q-Genie was published with a view to standardize CA appraisal in genetics [26].

Our study aims to document the choice, format and utilization of QA tools in SRs and meta-analysis in the field of human genetics. In the literature, terms such as quality assessment, quality appraisal, critical appraisal, and methodological quality are sometimes employed interchangeably [12]. In this manuscript, we use both quality assessment and critical appraisal, but we distinguish them from reporting quality.

## Methods

This methodology review was conducted in accordance with the Preferred Reporting Items for Systematic Reviews and Meta-Analyses (PRISMA) statement [5]. The protocol was registered with PROSPERO (CRD42023449349).

### Eligibility criteria

We included all SRs in human genetics, with or without meta-analyses. Intervention reviews, diagnostic test accuracy reviews, methodological reviews, qualitative reviews, epidemiological reviews, prevention reviews, pharmacogenetic reviews, and prognostic reviews were eligible. We included records identified by authors as systematic reviews, except for Cochrane reviews since these were presumed to adhere to Cochrane methodology and RoB tools. Studies were excluded if they (a) were not published in English or French, (b) were classified as books, comments, newspaper articles, editorials, letters, notes, conference abstracts, or protocols, (c) were overviews, or (d) focused on infectious diseases, dentistry or odontology, plants or agriculture, microbiota, sports, forensic sciences, genotoxicity, or in vitro studies.

### Search strategy

We searched the electronic databases MEDLINE, EMBASE, PubMed, and Web of Science. The search strategies included terms related to CA tools employed in SRs in human genetics. We excluded animal studies. The search was conducted, without time limits, up to May 19^th^, 2023, and updated on January 29^th^, 2024. The full search strategies are presented in Table S1.

### Study selection

We eliminated duplicates manually and transferred the search results to EndNote V20.6. Utilizing a random number generator from https://www.random.org, we sampled a 10% subset of these records and exported them to Rayyan (https://www.rayyan.ai). Subsequently, two reviewers independently screened the titles and abstracts for relevance, followed by a thorough examination of potentially eligible full-text articles. Throughout this process, reviewers remained blinded to each other’s decisions. Any discrepancies were resolved through discussion between the reviewers.

### Data extraction and synthesis

After thoroughly examining eligible full-text articles and online supplementary material, two reviewers independently conducted data extraction. The extracted information includes the first author’s name, country, year of publication, journal details (name, type, PRISMA endorsement or recommended PRISMA adherence), title, study topic, protocol registration, types of primary studies included in SRs, data synthesis methods, conflict of interest, funding sources, stated adherence to reporting guidelines, tools utilized for CA of primary studies, and presentation and use of CA results. The extracted data were recorded in a pretested Microsoft Excel spreadsheet. Any disparities in the extracted data were resolved through discussion between the reviewers and consultation with a third reviewer if necessary.

Our review primarily focuses on a specific methodological aspect of the included SRs, namely the use of CA tools. We therefore did not proceed with a comprehensive evaluation of the quality of the entire reviews.

We utilized descriptive statistics to outline frequencies and proportions for the extracted variables related to QA tools. Results were tabulated, including stratified results when relevant according to variables such as adherence to reporting guidelines or type of research question. SAS 9.4 was used to calculate confidence intervals (CI) (Clopper–Pearson exact method) and Chi-square tests. We did not conduct any meta-analysis, since our objective was not to produce pooled estimates for a given clinical question.

## Results

**Fig. 1:**
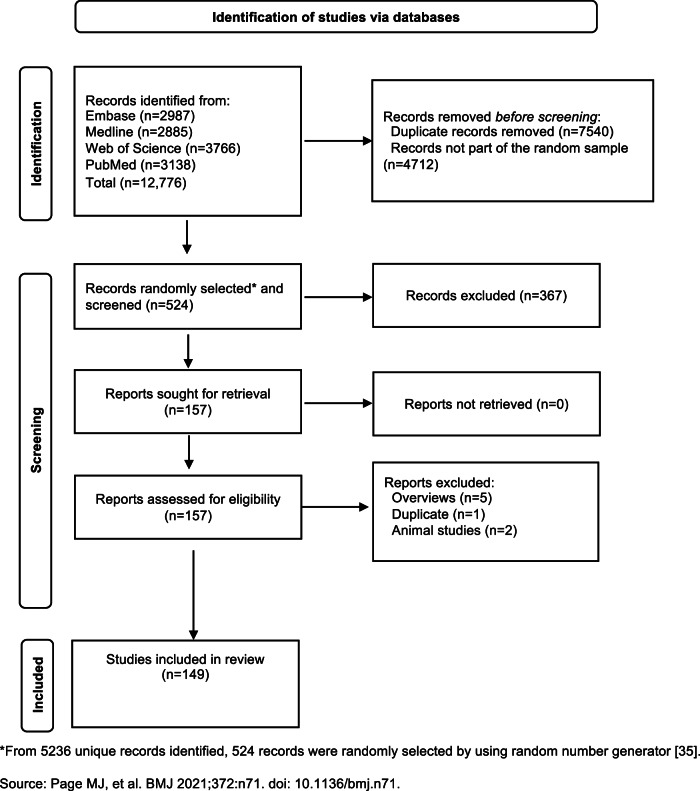
Flowchart for systematic reviews and meta-analyses.

The flow of articles through the screening process is presented in Fig. 1. We identified 5236 unique citations through the literature search. We randomly sampled 524 records for screening and selected 157 SRs for full-text review. Finally, we included 149 SRs (Table S2). A list of excluded articles and reasons for exclusion is available in Table S3.

### Basic features of included reviews

Over 75% of SRs were published between 2015 and 2024, and more than half (51.7%) originated from Asia, predominantly (47.7%) from China. Oncogenetics accounted for almost half of the reviews (45.6%). Twenty-three SRs (15.4%) were published by journals endorsing the PRISMA statement. Moreover, most journals (81.2%) recommended adherence to PRISMA guidelines. The authors of 35 reviews (23.5%) had registered their protocol, of which 33 with PROSPERO. Most SRs (87.2%) included a declaration regarding conflicts of interest. Only five (3.3%) explicitly acknowledged a potential conflict of interest. One-third of SRs received public funding, while funding sources were not specified in about 25%. Commercial funding sources were disclosed in only 2 SRs (Table 1).

**Table 1:** Features of included systematic reviews (*n* = 149).

Publication Characteristics	*n* (%)
Region	
Asia^a^	77 (51.7)
Europe	26 (17.5)
North America	10 (6.7)
Multicentric studies	19 (12.8)
Other	17 (11.4)
Health care field	
Oncology	68 (45.6)
Cardiology	11 (7.4)
Obstetrics	9 (6.0)
Neurology	8 (5.4)
Other^b^	53 (35.6)
Journal	
PRISMA endorsing	23 (15.4)
Recommending PRISMA adherence^c^	121 (81.2)
Protocol registered	35 (23.5)
Conflict of interest declaration	130 (87.2)
Conflict of interest declared	5 (3.3)
Funding sources	
Public	49 (32.9)
Commercial	2 (1.3)
Not‐for‐profit	7 (4.7)
Mixed	25 (16.8)
No funding	31 (20.8)
Not reported	35 (23.5)
Year of publication	
**≥** 2015	114 (76.5)
< 2015	35 (23.5)
**Methodological Characteristics**	**n (%)**
Guidelines mentioned	
Reporting guidelines	83 (55.7)
Reporting and methodological guidelines	10 (6.7)
Methodological guidelines	2 (1.3)
None mentioned	54 (36.3)
Number of research question treated	
1	135 (90.6)
2	12 (8.0)
3	1 (0.7)
4	1 (0.7)
Data synthesis method	
Quantitative	115 (77.2)
Narrative only	34 (22.8)
Type of primary studies included	
RCT	7 (4.7)
NRS	121 (81.2)
RCT and NRS	13 (9.4)
Economic studies	3 (2.7)
Mathematical modeling	4 (3.4)
RCT, NRS, Economic and mathematical modeling	1 (0.7)
CA tools identified in methods section	
Yes	136 (91.3)
Single CA tool	118 (79.2)
Multiple CA tools	18 (12.1)
No	13 (8.7)

^a^71 (47.7%) articles are from China.

^b^Other specialties represent less than 5% each.

^c^Six journals via the EQUATOR Network.

A quantitative data synthesis was conducted for at least one research question in 115 SRs (77.2%), whereas 34 SRs (22.8%) solely comprised a narrative synthesis. Many SRs (81.2%) included only non-randomized studies (NRS), while 13 reviews (9.4%) included both randomised controlled trials (RCTs) and NRS, and less than 5% only RCTs (Table 1).

### Adherence to reporting guidelines

**Fig. 2:**
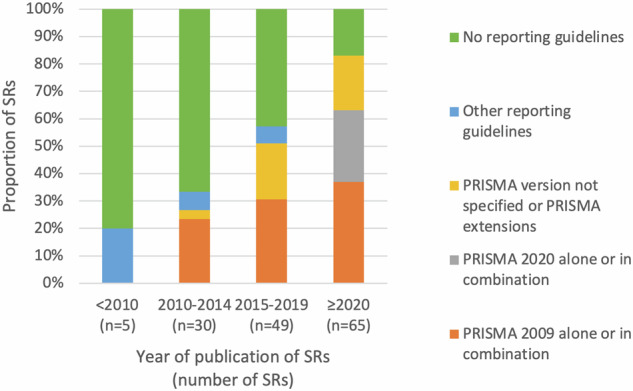
Distribution in time of self-reported adherence to various reporting guidelines. The proportion of each category is shown by various colors for SRs grouped according to their year of publication.

The authors of 93 reviews (62.4%) stated following reporting guidelines for SRs, two resorted to methodological guidelines, and 54 studies did not mention adhering to any guideline (Table 1). The evolution over time of self-reported adherence to reporting guidelines is illustrated in Fig. 2. The PRISMA and MOOSE [27] reporting guidelines were the most utilized, either alone (79.6%) or in combination with other guidelines (20.4%) (Table S4). The PRISMA checklist was only accessible for 22 SRs, including one SR that referred to MOOSE. In addition, a journal-specific reporting checklist, and the MOOSE checklist were filled out once each. Among the 23 SRs published in a PRISMA-endorsing journal, five (22%) did not mention using any specific reporting guideline.

Overall, three methodological guidelines, the Cochrane Handbook, CRD’s guidance, and the HuGE Reviews Handbook were cited in 11 SRs. Two authors stated following only methodological guidelines.

### Research questions

Most of the 149 included SRs (90.6%) addressed a single research question, 8% two research questions, and less than 1% three or four, which brought the total number of questions studied to 166. We classified research questions as follows: 1) Association studies, which investigated the association between biomarkers and disease risk or clinicopathological features of disease; 2) Diagnostic studies, which predominantly focused on diagnostic (or screening) accuracy, but included a few studies investigating diagnostic yield or the proportion of uninterpretable or unsuccessful tests; 3) Prognostic studies, which explored associations between risk factors and future health outcomes in patient populations; 4) Effectiveness of interventions, including one study that examined the effectiveness of screening strategies; 5) Effectiveness of genetic biomarkers, primarily focussing on predicting treatment response, such as chemoresistance or adverse reactions, but in two instances also analyzing the impact of using these biomarkers on survival; 6) Economic analyses; and 7) Prevalence studies.

Across all research questions (*n* = 166), half were categorized as association studies, with 13.3 and 15.1% as diagnostic and prognostic questions, respectively. Other research questions accounted for less than 10% each. Economic analyses were the least represented (Table 2).

**Table 2:** Type of research questions.

	Among articles addressing a single research question	Among all research question
Research question	*n* (%)	*n* (%)
Association studies	74 (54.8)	82 (49.4)
Diagnostic studies	18 (13.3)	22 (13.3)
Prognostic studies	16 (11.8)	25 (15.1)
Effectiveness of interventions	11 (8.1)	12 (7.2)
Effectiveness of genetic biomarkers	9 (6.7)	15 (9.0)
Economic Analyses	3 (2.2)	4 (2.4)
Prevalence studies	4 (3.0)	6 (3.6)
**Total**	**135**	**166**

### Critical appraisal tools

In their methods sections, 136 reviews reported using at least one CA tool, while in 13 (8.7%; 95%CI: 4.7–14.5%) none was mentioned (Table 1). These 13 reviews included exclusively NRS, 9 relied on a quantitative synthesis and most concerned association studies. In 18 articles, more than one CA tool was listed, with different tools chosen for different research questions or for different primary study designs. This approach was not systematically adopted however. Indeed, a single CA tool was mentioned in 17 reviews including both RCTs and NRS or several research questions, while several CA tools were listed in five reviews with a single research question and only one study design. Altogether, 156 references to CA tools were found across 136 articles (Table 3).

**Table 3:** Critical appraisal tools used, amongst all systematic reviews and amongst those adhering to PRISMA, QUOROM, MOOSE, or CHARMS reporting guidelines.

	All SRs	SRs adhering to reporting guidelines	
CA Tools	*n* (%)	*n* (%)	Tools referenced in SRs
**Generic tools:**			
NOS	57 (36.5)	38 (37.6)	[42]
QUADAS and QUADAS-2	18 (11.5)	10 (9.9)	[28]
Cochrane’s RoB and RoB-2	13 (8.3)	11 (10.9)	[3, 29]
Tools for RCT and NRS	10 (6.4)	9 (8.9)	Modified JADAD scale [43], ROBINS-I [14], ACROBAT-NRSI [44], AXIS [45], JBI critical appraisal tools [46] NIH-case-control [47]
Tools for economic studies	7 (4.5)	5 (5.0)	Drummond & Jefferson [48], Philips checklist [49], QHES [50], ISPOR-AMCP-NPC [51]
Tools for other study types	6 (3.8)	5 (5.0)	EPHPP [52], MMAT [53], QUIPS [54], PROBAST[55]
**Customized tools:**			
For genetic association or for pharmacogenetic studies	14 (9.0)	6 (5.9)	Thakkinstian 2004/2005 [56, 57], Clark & Baudouin 2006 [58], Maruthur 2014 [59], Amanat 2020 [60]; Q-Genie [26]
Tools inspired by reporting guidelines	14 (9.0)	9 (8.9)	STROBE [24], STARD [61], MOOSE [27], REMARK [62], STREGA [23]
Tools developed in-house	8 (5.1)	2 (2.1)	—
Content-specific and other tools	9 (5.7)	6 (5.9)	Tools adapted from Longnecker 1988 [63], Schlosser 2014 [64], Sotiriades 2007 [65]; RQS [66]; Dutch Cochrane Centre [67]; JBI [68] and Oxford Centre levels of evidence [69]
**Total number of CA tools**	**156**	**101**	

*ACROBAT-NRSI* A Cochrane Risk Of Bias Assessment Tool-for Non-Randomized Studies of Interventions, *AXIS* Appraisal tool for Cross-Sectional Studies, *EPHPP* Effective Public Health Practice Project, *ISPOR-AMCP-NPC* International Society for Pharmacoeconomics and Outcomes Research - Academy of Managed Care Pharmacy - National Pharmaceutical Council, *JBI* Joanna Briggs Institute, *JBI-PCAT* Joanna Briggs Institute-Prevalence Critical Appraisal Tool, *MMAT* Mixed Methods Appraisal Tool, *MOOSE* Meta-analysis Of Observational Studies in Epidemiology, *NIH-case-control* National Institutes of Health-case-control, *PROBAST* Prediction model Risk Of Bias ASsessment Tool, *Q-Genie* Quality of the genetic association studies, *QHES* Quality of Health Economic Studies, *QUIPS* Quality In Prognosis Studies, *REMARK* Reporting Recommendations for Tumor Marker Prognostic Studies, *ROBINS-I* Risk Of Bias In Non-randomized Studies - of Interventions, *RQS* Radiomics Quality Score, *STARD* Standards for Reporting Diagnostic Accuracy Studies, *STREGA* Strengthening the Reporting of Genetic Association Studies, *STROBE* Strengthening the Reporting of Observational Studies in Epidemiology.

**Fig. 3:**
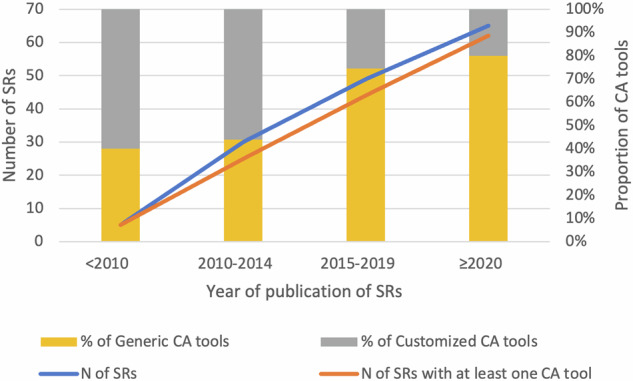
Trends in time for the total absolute number of SRs (blue line; left axis) and number of SRs using at least one CA tool (orange line; left axis). The histogram bars and right axis show the proportion of generic (yellow) versus customized (grey) CA tools.

We distinguish generic tools, i.e. those intended for specific study designs or study types, such as diagnostic or prognostic studies, from tools customized for a particular clinical domain, such as genetics, developed ad hoc, or adapted from reporting guidelines. Nineteen different generic CA tools accounted for 111 out of 156 citations (71.2%; 95%CI: 63.4–78.1%), whereas customized tools, usually unique, were recorded 45 times. The proportion of generic tools used increased in recent years (Fig. 3).

### Generic tools

The generic CA tools most frequently referred to are the Newcastle-Ottawa scale (NOS), the Quality Assessment of Diagnostic Accuracy Studies tool (QUADAS/QUADAS-2) and the Cochrane Risk-of-Bias tool (RoB/RoB-2), counting respectively for 36.5, 11.5 and 8.3% of citations.

NOS was used almost exclusively to assess NRS, most often in the context of association studies. In 51 of 57 citations, the reviews addressed questions of association, prognosis, prevalence or a combination thereof, while the effectiveness of pharmacogenetic markers was examined in five. In two SRs, NOS was used for controlled trials, but either only one arm of the trial was considered or the focus of the review was on a different contrast than that of the original trials.

The QUADAS and QUADAS-2 tools [28] were cited in 18 reviews, 15 of which involved diagnostic studies. They were also used in two reviews of association studies, and in one prognostic study which included both RCTs and NRS; all other reviews including only NRS. In four other reviews of diagnostic studies, CA was based on STARD, STROBE, the Joanna Briggs Institute (JBI) checklist for cohort studies or a modified version of the Cochrane RoB tool, whereas two reviews combining diagnostic and prognostic outcomes used NOS and PROBAST.

The Cochrane RoB and RoB-2 tools [3, 29] were cited in 13 SRs. They were used in 10 out of 12 reviews on the effectiveness of interventions (83.3%), the other interventional reviews mentioning ACROBAT-NRSI and an in-house developed tool. In another review focussing on association and prognostic studies, RoB/RoB-2 served to evaluate the RCTs. However, they were also used, albeit in an amended or partial form, in reviews of prognostic studies and prevalence studies, both of which only included NRS. Among 21 reviews including RCTs, 11 employed the Cochrane RoB/RoB-2 tools (52.4%).

The modified generic JADAD scale for RCTs was mentioned once and various tools once or twice for the evaluation of NRS: ACROBAT and ROBINS-I for interventions, AXIS for cross-sectional studies, the NIH tool for case-control studies, and JBI quality checklists. In each instance, the choice of tool was coherent with the research question addressed.

The following tools for assessing economic studies were used in the context of narrative syntheses: the Drummond & Jefferson checklist, the Philips checklist, and QHES. One article, with a strong focus on the quality of economic studies, cited all three. The ISPOR-AMCP-NPC Questionnaire was used in one review to evaluate mathematical modeling studies, whether they included clinical or economic outcome measures.

The remaining generic tools comprise multi-design tools, such as MMAT and EPHPP, and tools for prognostic or prediction model studies, namely QUIPS and PROBAST. The six reviews citing these tools either addressed research questions around prognosis or included modeling studies.

### Customized tools

Customized tools in the field of genetics include tools originally developed by authors for their own use in the context of genetic association studies and subsequently adapted by other authors for analogous studies. Some of these tools contain items tailored for specific clinical problems. Several underwent many modifications before being again adapted by the authors of the papers selected for the current review. More recently, the Q-Genie tool was developed for standardizing the CA of genetic association studies in SRs. This category also includes two tools elaborated for pharmacogenetic studies. Only one review citing this group of tools investigated the effectiveness of a pharmacogenetic marker. Most were classified as association studies and relied on NRS.

In 14 reviews, tools inspired by reporting guidelines were cited as CA instruments. Reporting guidelines alone served as CA tool in nine SRs. The information provided on the retained criteria was usually very restricted, particularly for STARD, REMARK and MOOSE, and, even for STROBE and STREGA, the number of items varied between 4 and 27. Tools inspired by STARD and REMARK were used exclusively for diagnostic and prognostic studies respectively, while the others dealt with association, pharmacogenetic intervention and prevalence studies.

Tools developed in-house included: 1) lists of 2 to 14 criteria for whom sources were not described, and 2) criteria assembled from a combination of different sources, including existing CA tools, reporting guidelines or methodological guidelines. Among the latter, one SR provided a list of over 20 items with a scoring system; two others provided very limited information on quality criteria, although study limitations were described.

The last category listed in Table 3 encompasses 1) tools adapted from existing tools for specific clinical problems, 2) classification schemes for levels of evidence and grades of recommendations based primarily on study design, and 3) tools for which no clear description was provided.

### Reporting guidelines adherence and choice of critical appraisal tools

Among the 93 SRs following PRISMA, MOOSE, QUOROM or CHARMS guidelines [30], a total of 101 CA tools were cited, while none was mentioned in 5 SRs (5.4%; 95%CI: 1.8–12.1%). The most cited CA tools included NOS (37.6%), Cochrane RoB/RoB-2 (10.9%), and QUADAS/QUADAS-2 (9.9%). Generic tools accounted for 77.2% (95%CI: 67.8–85.0%) of CA tools (Table 3). In the subset of 54 SRs that lacked adherence to any reporting guidelines, a total of 53 CA tools were documented, of which generic tools represented 60.4% (95%CI: 46.0–73.5%). It is worth highlighting that eight SRs did not mention any CA tools (14.8%; 95%CI: 6.6–27.1%). Further examination of 23 SRs published in a PRISMA-endorsing journal revealed the identification of 23 CA tools across 22 SRs. Of these, 16 CA tools (69.6%) were categorized as generic tools. No CA tool was mentioned in one SRs (4.4%).

### Presentation of critical appraisal results

For 90 of the 156 CA tools identified across 136 SRs (57.7%; 95%CI: 49.5–65.6%), meticulously detailed results for each study were provided, item by item or domain by domain. In contrast, for 43 CA tools (27.6%) results were summarized globally per study, and results were aggregated across studies for 15 (9.6%) tools (Table 4). In three SRs that included eight CA tools, results were not reported (2.7% for the generic group and 11.1% for the customized group). By comparison, when examining 93 SRs adhering to standardized reporting guidelines, detailed results were reported for 63.4% (95%CI: 53.2–72.7%) of CA tools. Among the 54 SRs not following any reporting guideline, detailed CA tool results were provided in 47.2% (95%CI: 33.3–61.4%) of cases. Furthermore, among two articles referring to methodological guidelines only, one presented detailed results while the other aggregated them.

**Table 4:** Presentation of quality assessment results.

Presentation of results	All CA tools	Generic tools	Customized tools
	*n* (%)	*n* (%)	*n* (%)
Detailed per item or domain for each study	90 (57.7)	73 (65.8)	17 (37.8)
Global per study	43 (27.6)	27 (24.3)	16 (35.6)
Aggregated across studies	15 (9.6)	8 (7.2)	7 (15.6)
None	8 (5.1)	3 (2.7)	5 (11.1)
**Total number of CA tools**	**156**	**111**	**45**

We observed that NOS results were less frequently reported in detail (47.4%; 95%CI: 34.0–61.0%) than those of other generic tools, whose results were detailed in 85.2% (95%CI: 72.9–93.4%) of cases (Chi-square = 17.61, *p* < 0.0001). Results of the Cochrane RoB/RoB-2 tools were most frequently detailed (92.3%). Detailed results were reported for only 37.8% (95%CI: 23.8–53.5%) of customized tools, as compared to 65,8% (95%CI: 56,2–74,5%) of generic tools (Chi-square = 10.28, *p* = 0.0013).

### Utilization of critical appraisal tools

We examined whether CA tools were used: 1) in their original format, 2) in part, or 3) in an amended format, either leaving out items or modifying their labels. Minor modifications allowed by the tool developers or changes to the scoring system were not counted in the ‘amended’ category. We validated the groupings based on the results provided whenever possible. The format of CA tools was deemed non-verifiable when results were not detailed and insufficient information was provided in the methods section. The format was considered verifiable for 108 CA tools, of which 78 (72.2%) were applied in their original form. NOS (90.3%), QUADAS/QUADAS-2 (87.5%), and Cochrane RoB/RoB-2 (76.9%) stood out in this respect. Overall, 80.2% (95%CI: 69.9–88.3%) of generic tools figured in this category, against 48.2% (95%CI: 28.7–68.1%) of customized tools (Table S5).

Among 106 reviews featuring a quantitative synthesis for at least one research questions and citing at least one CA tool, QA results were utilized in 15.1% of cases for the selection of primary studies, in 21.7% in the analysis, and in 22.4% to explain the findings of the SR or meta-analysis. In contrast, among 30 SRs without meta-analysis citing at least one CA tool, authors of 6.7% reports used QA results for study selection, and 26.7% for explaining their findings (Table S6).

### Grading systems

In addition to the CA of individual primary studies, grading systems were used in 10 SRs to evaluate the credibility or certainty of the body of evidence. The grading systems mentioned were GRADE in eight SRs (5.4%) and VENICE in two SRs (1.4%). The latter system [31] was elaborated for cumulative epidemiologic evidence from genetic association studies.

### Association studies case-study

A detailed account of the characteristics of association studies is presented in Tables S7–S9. Among the 82 SRs (54,9%) including at least one such research question, 35 (42.7%; 95%CI: 31.8–54.1%) mention no guideline, 9 (11.0%; 95%CI: 5.1–19.8%) mention no CA tool and the majority (77 or 93.9%; 95%CI: 86.3–98.0%) include only NRS. Of the 79 citations of CA tools, 51 (64.6%; 95%CI: 53.0–75.0%) were generic and 28 (35.4%; 95%CI: 25.0–47.0%) were customized tools. Detailed results were presented for 54.9% (95%CI: 40.3–68.9%) of the former and 32.1% (95%CI: 15.9–52.4%) of the latter. For NOS, the most frequently used tool, the format of use was not verifiable in 41.9% (95%CI: 27.0–57.9%) of instances, but, when verifiable, it was used in its original format in 92.0% (95%CI: 74.0–99.0%) of cases.

## Discussion

The increasing number of systematic reviews published over the last decades accentuates concerns regarding the quality of these studies and raises questions about the formal training in research methodology among authors, peer reviewers and editors [10, 32, 33]. Landmark papers in the field are the cross-sectional studies on the epidemiological and reporting characteristics of SRs published in November 2004 [34] and in February 2014 [33]. More recently, similar studies reviewed SRs on the effectiveness of interventions that included NRS [15] or SRs on health state utilities [12]. In addition, two articles focussed on the planned use of appraisal tools in non-Cochrane SRs registered with PROSPERO [35, 36], while the adherence to the PRISMA 2020 Statement by recent non-Cochrane SRs on interventions was examined item by item [37].

In the field of human genetics, the number of published SRs has exploded, and to our knowledge, no review has systematically investigated their quality. This study aims to partially fill this gap by documenting the self-reported adherence to reporting guidelines and the choice and modalities of use of CA tools. While Page and colleagues found that 4% of SRs had registered a protocol with PROSPERO and that 29% of SRs used a reporting guideline [33], 23.5% of the SRs in our sample had registered a protocol and 81.2% reported adherence to PRISMA or other reporting guidelines. We found that at least one CA tool was mentioned in the methods section for 91.3% (95%CI: 85.5–95.3%) of all included SRs and for 94.6% (95%CI: 87.9–98.2%) of SRs with self-reported adherence to a reporting guideline. Previous estimates of risk-of-bias or quality assessment in SRs ranged from 55% [12] to around 70% [33, 34], but lower estimates were documented for SRs including NRS and for non-Cochrane SRs with 33% [17] and 50 to 57% [33, 34], respectively. Intended use according to registered protocols ranged from 80% [15] to 90% [35]. Ruano and colleagues excluded between 11 and 14% of identified PROSPERO records because of lack of information in the risk-of-bias assessment field [36] and, according to Ivaldi and colleagues, adequate reporting of the study risk-of-bias assessment in the PRISMA methods section lies on average well below 25% [37]. A grading system was deployed in 6.7% of SRs compared to 11% previously recorded [33].

Numerous methodological guidelines for the conduct of SRs and MAs, as well as the tools intended to evaluate the quality of SRs, AMSTAR/AMSTAR-2 and ROBIS, include indications as to the reporting of CA results and their use. The PRISMA Statements 2009 and 2020 emphasize that reporting only summary data is inadequate and that discussion of the validity of included studies is essential, while discussion of limitations of the review process itself is considered insufficient [5]. Detailed results per item or per domain were provided for 57.7% of the 156 CA tools listed, a proportion which rose to 63.4% when authors reported adherence to reporting guidelines. For association studies, the majority of results were presented in an aggregated or global form. Among 106 articles with a quantitative synthesis, 21.7% performed sub-group or sensitivity analyses clearly linked to the CA results. This does not mean that the remaining articles did not perform such analyses for other variables. For instance, Hardy Weinberg Equilibrium amongst controls was occasionally used for that purpose, even when this consideration was not part of the identified CA tool. Previous accounts estimated that QA was used to inform data analysis only in 5% [12] to 16% [33] of surveyed SRs. Adherence to the PRISMA 2020 item on presentation of results of risk-of-bias assessment for each included study approached 40%, whereas the item on discussion of limitations of the evidence received a score close to 90% [37]. In the present study, only 23.5% of discussions clearly referred to the results of CA to explain the limitations of included primary studies, which does not exclude that in the remaining SRs the limitations of the review process itself may have been mentioned. However, unless biases in primary studies are judged minimal, data quality concerns should be addressed when interpreting SRs’ findings and drawing conclusions [7].

Kolaski and colleagues underscore that RoB tools should not be modified without complete transparency [10]. In our sample, few authors acknowledged using modified versions of the most common generic tools, but approximately 20% of generic tools and over 50% of customized tools appeared amended or used in part. Detailed information and justifications were rarely provided regarding the adopted modifications and occasionally crucial items, such as those pertaining to study design or risk of bias, were omitted despite extensive detail on less important considerations. Moreover, we did not tally among the observed amendments modifications made to scoring systems. A summary score was frequently computed for NOS, in over a quarter of instances for QUADAS, and even occasionally for tools inspired by reporting guidelines.

Among the generic tools, NOS was by far the most popular tool, as has been reported previously [15, 35]. Given NOS results are often presented per domain rather than per item, it was impossible to verify whether the appropriate version, for cohort or for case-control studies, had been used. Given the scope of study designs included under NRS, a more detailed examination of the correspondence between the study design and the choice of CA tool would be warranted. However, the information on study design was often very limited. While reference lists could be scanned to confirm or exclude the inclusion of RCTs, retrieving included publications to determine study design was beyond the scope of this work. The popularity of NOS, despite the fact that drawbacks have been highlighted [14, 38, 39] has been attributed to its ease of use [15, 35]. It should be noted however that facility perhaps also drives the further utilization of its results. Indeed, these are either not presented, presented in an aggregated format across studies or as a summary score per included primary study in over 50% of cases. Interestingly, roughly one-third of SRs utilizing NOS cite Stang’s 2010 publication as reference, without mentioning any limitations [38, 40].

The context of use for the Cochrane RoB/RoB-2 tools and for QUADAS/QUADAS-2 was coherent with their intended use in a majority, though not in all cases. The former were occasionally employed for NRS. As pointed out by Quigley and colleagues, tools for RCTs are unlikely to be appropriate for NRS which are vulnerable to a wider range of biases [15]. Conversely, in the field of human genetics, SRs on prognosis or on efficacy of pharmacogenetic markers sometimes use data from single arms of RCTs or data extracted from studies with different contrasts than that addressed by the SR. This raises questions about how items on randomization for instance are being interpreted in such circumstances and on the choice of the most appropriate CA tool. Likewise, for diagnostic research questions wherein individuals cannot systematically receive an index and a reference test, such as happens in prenatal care, the use of QUADAS may pose difficulties with regard to some items. The remaining generic tools were usually applied in appropriate contexts, the more recently developed ones being presumably underused. For instance, although four SRs included mathematical modeling studies, PROBAST was mentioned only once.

In 2016, Page and colleagues reported that 18% of SRs used “author-developed” tools. In the present study, customized tools represent 28.8% (95%CI: 21.9–36.6%) of all CA tools and 22.8% (95%CI: 15.0–32.2%) of those used when authors reported following reporting guidelines. Several reporting guidelines contain explicit warnings against their use as QA tools (PRISMA, STREGA, and STROBE). Yet, tools inspired by reporting guidelines represented 9% of cited tools, which is comparable to the 9.4% intended use of “reporting quality” found by Ruano and colleagues [36]. Tools developed in house counted for 5.1% in our sample. Of note, 34% of the CA tools for observational studies examined by Sanderson and colleagues were intended for a single use in a specific SR [16]. The choice to develop or add on an in-house tool was sometimes motivated by the desire to include in the appraisal particular technical aspects related to relevance or quality. Some generic tools, such as QUADAS-2 and QUIPS, provide room for the operationalisation of signalling questions to address such concerns, but this option is not always available. Of note, in two SRs, a level of evidence and a grade of recommendation, based on the JBI and the Oxford Centre schemes, were attributed to every primary study. Kolaski and colleagues stress that the use of hierarchical levels of evidence based on study design only, without analysis of potential biases and other shortcomings, is outdated and that tools developed for CA of primary studies need to be distinguished from tools involved in appraising the certainty of the body of evidence [10].

Opting for a customized tool could result from unfamiliarity with existing or recent tools but could also be a response to perceived unfulfilled needs in a particular epidemiological or clinical situation [41]. The lack of appropriate tools in the field of human genetics was noted in 2015 [32], although several initiatives to adapt or construct specific tools accrued since 2000. No single tool seems to have been widely adopted. We observed that 9% of tools were intended for genetic association or for pharmacogenetic studies. Even when singling out the association studies, these represented but 15.2% of tools and Q-Genie was used only twice. In addition, the STREGA reporting guideline and the HuGENet Review Handbook constituted sources of inspiration for the CA appraisal process in several SRs. One of the common problems with customized tools is the lack of justification for the inclusion of specific items [18]. In addition, their validity and reliability has usually not been studied and the absence of instructions hampers a reproducible use of such tools. We show that customized tools results were detailed significantly less often than for generic tools. Yet, they were utilized for the selection of primary studies, in the analysis, or for the interpretation of findings in 48.9% (95%CI: 33.7–64.2%) of cases versus 36.9% (95%CI: 28.0–46.6%) for generic tools.

### Strengths and limitations

To our knowledge, we conducted the first systematic methodology review of tools used to appraise the quality of studies in human genetics, a domain with ramifications in most health care fields. The strengths of our study are that 1) we registered the protocol in PROSPERO, 2) we implemented a comprehensive search strategy in multiple databases, 3) two reviewers independently selected studies and conducted data extraction, 4) we examined the presentation of CA results and their subsequent use by authors in greater detail than other published reviews, and 5) most SRs (76.5%) in our sample were published after 2015.

Our study has some limitations: 1) we did not search any grey literature and may thus have missed eligible reviews, 2) we randomly selected a subset of SRs, 3) we opted not to systematically include the 95% confidence intervals in the tables to avoid overloading them, 4) we restricted our search to SRs in French or English, and 5) we conducted data extraction solely from full-text SRs and online supplementary material, without contacting the authors.

The quality of the information provided by SRs’ authors displayed several limitations, some of which had implications for our capacity to analyse in depth the adequacy of their QA efforts. For instance, several authors failed to clearly state which study designs they considered eligible and did not include this crucial information when presenting basic data on primary studies. CA tools were frequently used in amended or partial formats without justification for these modifications, but this could only be appraised with a sufficiently detailed description of the tool or detailed results. Occasionally, all primary studies did not undergo quality assessment, without explanations thereto. Finally, the latest versions of CA tools were not always used despite their availability at time of publication.

In conclusion, this systematic methodological review of the appraisal tools used for evaluating the quality of primary studies within SRs in the field of human genetics uncovered a total of 156 citations of CA tools across 149 papers. The proportion of authors reporting adherence to reporting guidelines was higher than that reported in previous publications. However, as previously reported [10], self-reported adherence to guidelines does not always translate into superior methodological rigor. In our sample, a number of encouraging trends were observed, with less SRs omitting QA entirely, a greater use of generic tools and more detailed reporting of CA results in the group of SRs with self-reported adherence to reporting guidelines than in the group of non-adherers. However, these differences did not reach statistical significance. A completed PRISMA checklist was only available online for less than a quarter of self-reported adherers. Moreover, several shortcomings need to be highlighted, including a lack of transparency with regard to study design, which is a prerequisite to the choice of an appropriate CA tool [10, 32], a lack of justifications regarding the choice of CA tools and of any amendments thereof, as well as deficiencies in the presentation of CA results and limited use of CA results. Of particular concern, detailed results were available for less than half of the customized tools, as well as for NOS. This observation is equally valid for the association studies. Overall, among the 136 reviews having listed at least one CA tool, less than a quarter presented detailed results for at least one CA tool and used the results of the appraisal either for the selection of primary studies or for the analysis or interpretation of their findings. These shortcomings in the CA of primary studies have the potential to undermine the validity of the findings and conclusions of SRs in human genetics. Warnings have recently been issued against superficial use of reporting guidelines [10]. Our study suggests that superficial use of CA tools, perhaps particularly user-friendly ones, might also be an issue.

To improve the quality of published SRs, Kolaski and colleagues produced a concise guide of best practices to ‘promote understanding of the demanding science of evidence synthesis’ among authors, peer reviewers and editors [10]. A similar endeavour could be developed for common study designs in the field of genetics. Ivaldi and colleagues stress the need to allocate funds and build capacity within research teams [37], an avenue demanding widespread and consequential efforts. In the short term, a notable improvement could nevertheless be achieved if, whenever applicable, peer-reviewers and editors required that information on study design, choice of CA tool, results of QA and subsequent use thereof be made systematically accessible.

## Supplementary information

Table S1

Table S2

Table S3

Table S4

Table S5

Table S6

Table S7

Table S8

Table S9

PRISMA ABSTRACT checklist

PRISMA checklist

## Data Availability

The dataset and/or analyses are available from the corresponding author on reasonable request.
