# Supplementary figures and images for: Tools used to appraise the quality of studies included in systematic reviews and meta-analyses in human genetics: a systematic review
Source: Eur J Hum Genet. 2025 May 21;33(11):1392–401. doi: 10.1038/s41431-025-01861-6 (PMC12583517; doi:10.1038/s41431-025-01861-6)

**Supplementary materials**


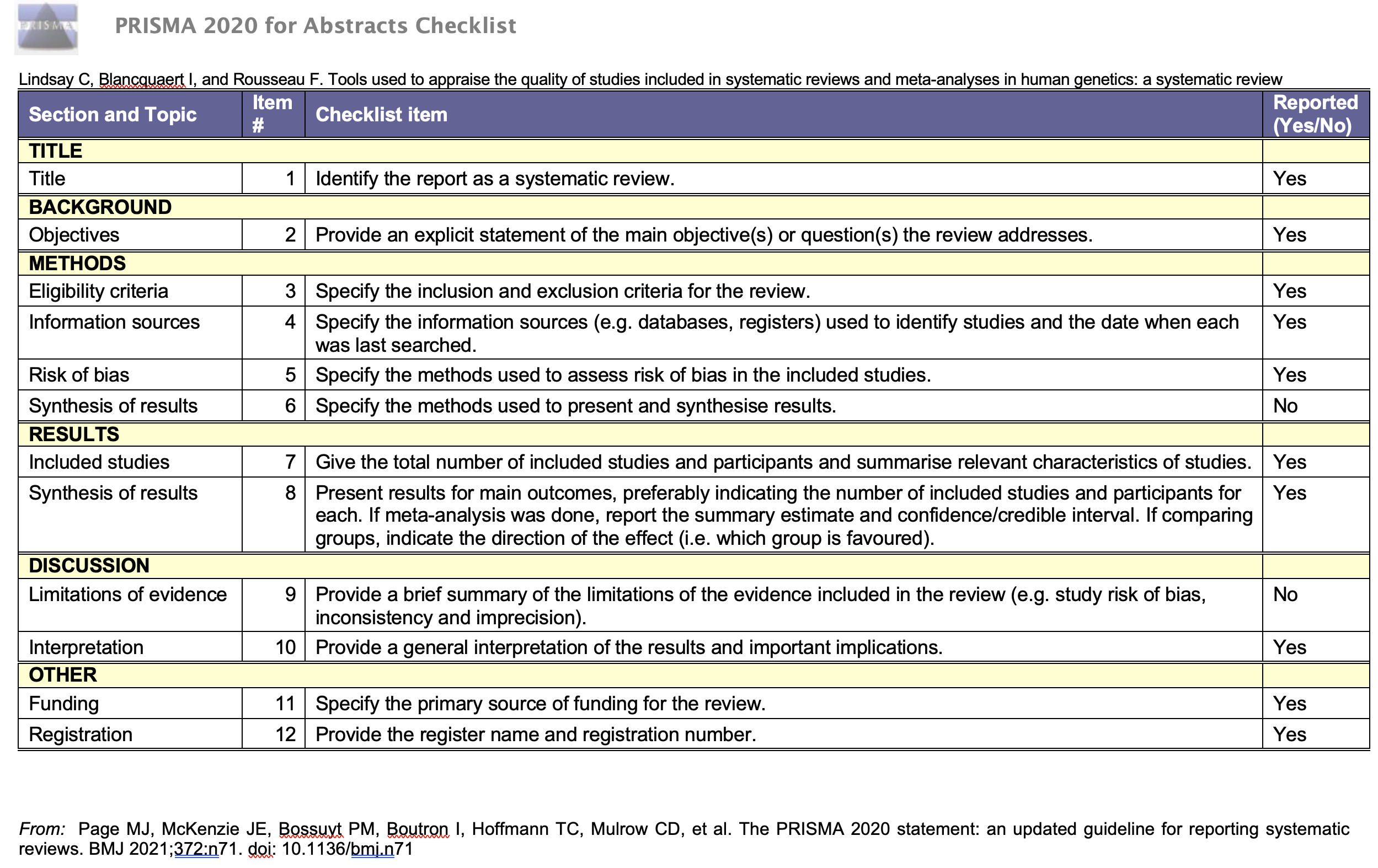

Supplement: Supplementary file 10 — PRISMA ABSTRACT checklist [file 41431_2025_1861_MOESM10_ESM.docx]
